# Valproate in Conversion Disorder: A Case Report

**DOI:** 10.1155/2010/205702

**Published:** 2010-06-30

**Authors:** Antonino Messina, Anna Maria Fogliani

**Affiliations:** Medical and Surgical Specialties, School of Psychiatry, Faculty of Medicine and Surgery, University of Catania, Via S. Sofia, 78, 95123 Catania, Italy

## Abstract

Few data are in literature about the pharmacological treatment of conversion disorder and there are not any studies about the use of Valproate extended release (ER) in treating conversion disorder. In this article, we are reporting a case of an Italian woman with a diagnosis of conversion disorder treated effectively and quickly by Valproate ER.

## 1. Introduction

The presence of neurological signs, as deficits of sensitive or motor activity, without any organic lesions, is actually diagnosed as conversion disorder (DSM IV-TR) [1]. The term conversion introduced by Sigmund Freud [2] and used as a defence mechanism of the Ego, substituted the diagnosis hysterical neurosis in DSM III (1980) [3]. 

 Although the term hysteria comes from a Greek word, meaning uterus, the ancient concept of hysteria was different from the current one [4]. Ancient Greek described a syndrome characterized by numerous symptoms as stifling, aphonia, heart palpitation and other somatic symptoms, but the hypothesis of unspecified behavioural disturbances, caused by a displacement of the womb or by a “wandering uterus” were not reported in ancient Egyptian nor in Greek texts [5]. Hippocrates described the role of the womb in the hysterike pnix, traduced as stifling, but the Hippocratic concept of hysteria was not linked to behavioural alterations [6]. 

 During the Middle Ages the magic and superstitious vision was predominant in medicine and the “psychopathology” of women with mental disorders, considered as witches, was described in the Malleus Maleficarum [7] (The Hammer of Witches, 1486), a textbook that reported “every type of neurosis or psychosis which we find today in our daily psychiatric work” [8]. 

 After the superstitions linked to hysteria were gradually abandoned and in Modern Age, Charles le Pois, for the first time, and others as Thomas Willis and Thomas Sydhenam considered hysteria to have a cerebral cause and later Russell Reynolds thought that some forms of paralysis could be caused by an idea [9]. 

 The association between a psychic disorder and the somatic manifestations was confirmed by Jean Marie Charcot who considered hysteria as a psychic disease “par excellence” [10]. During his leçons du mardi (Tuesday lessons), Charcot attracted many world physicians at Salpêtriere, to show the phenomenology of grande hysterie with the body characteristic phenomenology as the arc en cercle (extreme opisthotonus), the grandes movements and the attitudes passionelles (hallucinatory phase) [11]. Then in Freudian theory, hysteria was seen as a consequence of a conflict between unacceptable impulses, desires, thoughts and moral instances [12]. The libido linked to repressed memories of traumatic experiences or repressed sexual fantasies could be converted into somatic symptoms [12]. 

 Despite the fact that the libido hypothesis, as cause of hysteria, has been rejected by authors who dispute the psychoanalytic interpretation of psychiatric disease [13–15], the hypothesis of an intrapsychic conflict could be considered as ”an useful organizing concept” [16] and a combined neuroscientific-psychoanalytic approach could be useful to investigate the neurophysiopathology of mental disorders [17, 18] as well as the hysteria, however “there is no generally accepted explanation for how a psychological stress can change into (often highly selective) symptoms” [19]. 

 Actually functional neuroimaging researches have evidenced some cortical dysfunctions as a reduced activity in motor, sensitive and visual cortical areas during hysterical paralysis, hysterical anaesthesia and hysterical blindness [20]. 

 Hysteria remains the “bete noir” of medicine, the diagnosis is often uncertain and of exclusion without a specific pharmacologic therapy. 

 In this article we describe a case of a woman with conversion disorder treated with a pharmacological approach.

## 2. Case

A 34 years old woman was taken to the emergency room for a loss of consciousness and an absence of stimulus-response (attaques de sommeil [21]). No evidence of objective basis were found after instrumental exams and specialistic consults; this clinical condition continued for 48 hours.

 When the woman became conscious, she said not to remember anything about the event. She was discharged from the emergency room and she was admitted in our operative unit of psychiatry. She reported that for several months she has been experiencing a loss of self control (e.g. she cooked or remade the bed “without awareness”); these episodes of “loss of awareness” lasted few minutes (absences [21]). She also experienced dysperception as dysmegalopsia and eritropsia. On a psychic exam she showed no psychopathological signs, except a mild depressive status, a lack of concern and feeling and an indifference about her condition (belle indifference [21]). During the hospitalization she showed aphonia and astasia-abasia. Laboratory test, CT, NMR, EEG were normal. On psychological test, MMPI evidenced the presence of the so called “Conversion V”, a condition in which depression has lower score than hypochondrias and hysteria [21]. 

 According to DSM-IV-TR [1] the diagnosis was conversion disorder, non epileptic seizures subtype

 She was treated with Citalopram at 20 mg/die, that induced an improvement in depressive symptoms and a slight improvement in conversion symptoms. After 10 days she was discharged.

 Two weeks later she was seen again for a control visit, while she was talking about the recrudescence of symptoms, she suddenly was unable to speak, to stand up and to walk. This symptomatology was acutely treated with Lorazepam 2.5 mg per os and it remitted after 2 hours. The treatment was modified: Citalopram 20 mg/die was suspended, and after a written informed consent, Valproate extended release (ER) 300 mg/die was added. After the addition of Valproate ER the conversion symptoms rapidly and significantly improved and after one year of follow up she did not have any symptoms, she didn't lose consciousness and she did not show aphonia or astasia-abasia.

## 3. Discussion

The absence of objective basis of the symptomatology excludes the presence of an organic disease. The presence of neuropsychiatric signs, as astasia-abasia, aphonia, dysperception and crepuscular state, without organic lesion and electric abnormalities of brain to EEG, suggests the diagnosis of a conversion disorder. The presence of psychological difficulties (conflicts with her husband), associated to“Conversion V” on MMPI [22] confirms the diagnosis. No data can be found in literature about the use of Valproate in conversion disorder. A Previous research reported the use of lithium in conversion symptoms [23]. The mechanisms of action of lithium and Valproate are similar. Both drugs are inhibitors of *voltage*-dependent sodium and calcium channels, of glutamate neurotransmission and of second messengers (protein kinase C, inositol triphosphate and diacylglycerol), while both drugs potentiate GABA neurotransmission [24]^.^ The interpretation of action of Valproate on conversion disorder is unclear. Stonnigton and coll. [25] reported the utility of the assumption of antiepileptic drugs in conversion patients with no epileptic seizures associated with epilepsy, but in our case the presence of epilepsy was excluded. 

 Despite some data in literature evidenced that GABAergic anticonvulsant could induce pseudoseizures [26, 27], the use of Valproate ER in no epileptic subjects is well tolerated and it is not associated to a high incidence of side effects as pseudo epileptic seizures [28–32].

 This article shows that the addition of Valproate ER in therapy determined a remission of conversion symptoms. After Valproate assumption the patient didn't have episodes of alteration of consciousness and did not show aphonia, abasia-astasia. 

 We argue that the use of Valproate ER could accelerate and maintain the remission of hysterical symptomatology. Further researches on a larger sample need to investigate on the efficacy of Valproate ER in treating patients with conversion disorder, and to verify also if the use of Valproate ER could facilitate a more rapid resolution of symptoms.
